# Differential Interactions of Tissue-Restricted Host Proteins SPLUNC1 and VAMP8 with VP3 of Human Bocaviruses 1 and 2

**DOI:** 10.3390/pathogens15050486

**Published:** 2026-05-01

**Authors:** Ri De, Yanpeng Xu, Hanhaoyu Fu, Liping Jia, Linqing Zhao

**Affiliations:** 1 Laboratory of Virology, Capital Center for Children’s Health, Capital Medical University, Capital Institute of Pediatrics, Beijing 100020, China; graceride@163.com (R.D.); yanpxbj@163.com (Y.X.); fuhanhaoyu@aliyun.com (H.F.); im_jiaping@126.com (L.J.); 2 Graduate School of Peking Union Medical College, Beijing 100730, China

**Keywords:** human bocavirus 1, human bocavirus 2, tissue tropism, SPLUNC1, VAMP8

## Abstract

**Background:** Four genotypes of human bocaviruses (HBoVs) have been identified, with only HBoV1 being detected in respiratory specimens, and with HBoV2 being the predominant human bocavirus in fecal specimens, which implies different tissue tropisms for HBoV1 and HBoV2. It is vital to determine the factors that influence the tissue tropisms. **Methods:** The major capsid proteins VP3 of HBoV1 and HBoV2 were expressed in eukaryotic cells. Then co-immunoprecipitation (Co-IP) and liquid chromatography–tandem mass spectrometry (LC-MS/MS) (IP-MS) was employed, along with Gene Ontology (GO) and Kyoto Encyclopedia of Genes and Genomes (KEGG) analyses, to screen host proteins interacting with VP3 of different genotypes. Subsequently, in vitro pull-down assays were conducted to verify the direct virus–host interaction proteins with VP3. Furthermore, molecular docking was performed to predict the interaction interfaces between viral and host proteins. **Results:** Through IP-MS and enrichment analyses, 50 host proteins that displayed ≥10-fold differential binding affinities between HBoV1 VP3 and HBoV2 VP3 were identified. Among these, seven were considered as high-confidence candidate interactors. Notably, SPLUNC1 and VAMP8 showed predominant expression in respiratory and intestinal tissues, respectively. Subsequent in vitro pull-down assays confirmed that SPLUNC1 specifically bound to HBoV1 VP3, whereas VAMP8 specifically interacted with HBoV2 VP3. Molecular docking analysis further revealed that the binding between SPLUNC1 with HBoV1 VP3, as well as VAMP8 with HBoV2 VP3, was stabilized by extensive hydrophobic interfaces along with specific hydrogen bonds. **Conclusions:** The specific interactions of host proteins SPLUNC1 with HBoV1 VP3 and VAMP8 with HBoV2 VP3, respectively, provided fundamental evidence that the distinct tissue tropisms of HBoVs may be governed by specific host factors.

## 1. Introduction

Human bocavirus (HBoV) is a small, non-enveloped DNA virus belonging to the genus *Bocaparvovirus* and the family *Parvoviridae*. It has been categorized into four genotypes (HBoV1–4) since its initial discovery in 2005. HBoV1 was the only HBoV detected commonly in respiratory specimens from pediatric patients with acute respiratory infections (ARIs), and it has been confirmed to be a real etiological agent of ARIs ranging from the common cold to bronchiolitis and pneumonia [1,2,3,4]. Other human bocaviruses, HBoV2–4, were first identified in stool specimens from children with acute diarrhea in 2009 and 2010, respectively [5,6,7,8], but their causal role in gastrointestinal disease is uncertain, with HBoV2 being the most common finding in fecal specimens from children suffering from acute gastroenteritis, with the detection of viral particles and mRNA in the gut mucosa, which suggested the primary role of HBoV2 in enteric infections [9,10,11,12,13]. This compelling epidemiological dichotomy strongly suggested that HBoV1 and HBoV2 have evolved different tissue tropisms.

The molecular mechanisms governing viral tissue tropism are often orchestrated by the virus outer capsid proteins, which mediate the initial and critical steps of viral infection [14]. The linear single-stranded DNA genome of HBoVs encodes nonstructural proteins NS1–NS4 and NP1, and structural capsid proteins VP1–VP3. These capsid protein VPs share a conserved C-terminal domain and are expressed in an approximate ratio of 1:1:10, facilitating their self-assembly into non-enveloped, icosahedral virions [15]. In particular, VP3 expressed solely can autonomously form virus-like particles (VLPs). The expressed VP3 exhibited high immunogenicity and induced robust humoral and cellular immune responses [16]. It has been confirmed that there are four divergent regions (DR1–4) located on the surface of VP3 with different secondary and tertiary structures between HBoV1 and HBoV2. And antibodies against DR2, located on VP3 of HBoV1 or HBoV2, respectively, have been found to be genotype-specific [17,18]. The observed clinical divergence between HBoV1 and HBoV2 strongly suggests that their VP3 proteins have evolved distinct interaction profiles with host cellular factors. These differences could manifest as variations in receptor binding affinity, engagements with specific co-receptors, or interactions with intracellular proteins that facilitate viral replication in a tissue-specific manner. Therefore, it is a powerful and logical strategy to compare and analyze the host proteins that interact with the VP3 of HBoV1 and HBoV2 to reveal the molecular determinants of HBoV1 targeting respiratory and HBoV2 targeting intestinal tissue.

In this study, the molecular mechanisms underpinning the different tissue tropisms of HBoV1 and HBoV2 were preliminarily investigated by expressing the VP3 of HBoV1 and HBoV2 in Caco-2 cells, and excavating the genotype-specific VP3-interacting host proteins from the results of co-immunoprecipitation (Co-IP) and liquid chromatography–tandem mass spectrometry (LC-MS/MS) (IP-MS) combined with enrichment analysis of GO and KEGG. Then the in vitro pull-down assay was carried out to verify the virus–host direct interaction proteins and their affinity constant with VP3. Moreover, a molecular docking assay was performed to predict the interaction interfaces between virus and host proteins. Unraveling these virus–host interactions is crucial for developing a deeper understanding of HBoV pathogenesis and may open new avenues for the development of targeted antiviral strategies.

## 2. Material and Methods

### 2.1. Plasmids

The VP3 coding region sequences of HBoV1 (GenBank ID: No. DQ988934) and HBoV2 (GenBank ID: AFW98869.1) were cloned into the prokaryotic expression vector pET30b (Sangon Biotech Co., Ltd. (Shanghai, China)) with an N-terminal 6×His tag, and into the eukaryotic expression vector pcDNA3.1 (Beijing Bomaide Biotechnology Co., Ltd. (Beijing, China)) with a C-terminal StrepII tag. The integrity of all recombinant plasmids was verified by Sanger sequencing [19,20].

### 2.2. Expression and Purification of HBoV1 and HBoV2 VP3

The recombinant plasmids PET30b-HBoV1VP3 or PET30b-HBoV2 VP3 were transformed into *E. coli* BL21 (DE3) (Novagen, Madison, WI, USA) and induced by 1 mmol/L isopropyl-β-d-thiogalactopyranoside for 24 h. HBoV1 and HBoV2 VP3 were harvested from inclusion bodies with an N-terminal 6×His tag downstream of the T7 promoter, yielding proteins with the molecular weight of 70 kDa. After ultrasonication, the expressed VP3 was purified with nickel–nitrilotriacetic acid agarose (Qiagen, Germantown, MD, USA) following the manufacturer’s protocol [2,21].

### 2.3. Cell Culture and Transfection

Human colorectal adenocarcinoma cell line Caco-2 (Cell Bank of Peking Union Medical College) were cultured in Dulbecco’s Modified Eagle Medium (DMEM) supplemented with 10% fetal bovine serum (FBS) and 1% penicillin–streptomycin at 37 °C in a 5% CO_2_ incubator. Then pcDNA3.1-HBoV1VP3 or pcDNA3.1-HBoV2 VP3 were transfected into Caco-2 cells in 70–80% confluence using the Lipofectamine^TM^ 3000 Transfection Reagent (Invitrogen, Carlsbad, CA, USA), with the empty vector pcDNA3.1 as control, following the manufacturer’s protocol. After 48 h post-transfection, 1 × 10^7^ cells were collected and lysed for Western blotting (WB) or Co-IP assay.

### 2.4. Western Blotting (WB)

To ensure the successful transfection of pcDNA3.1-HBoV1VP3 or pcDNA3.1-HBoV2 VP3 by WB, Caco-2 cells collected after 48 h post-transfection were washed with cold PBS and lysed using a mild non-denaturing lysis buffer (Beijing Solarbio Science & Technology Co., Ltd., Beijing, China) containing protease inhibitors. Then HBoV1 and HBoV2 VP3 in the lysed cells were separated by 8–20% Sodium Dodecyl Sulfate–Polyacrylamide Gel Electrophoresis (SDS-PAGE) and transferred to nitrocellulose membranes (Invitrogen, USA). After blocking with 5% non-fat dry milk solution in TBST (Tris-Buffered Saline with Tween 20) at room temperature for 2 h, the membranes were incubated at 4 °C overnight with anti-HBoV1 DR2 polyclonal antibody, as previously described [18]. The next day, the membranes were washed three times in TBST and then incubated with horseradish peroxidase (HRP)-conjugated goat anti-rabbit IgG antibodies (Abcam, Cambridge, MA, USA) at room temperature for 1 h. The color was developed by addition of the Luminata Forte Western HRP Substrate, and the intensity of the immunoblot bands was quantified using a System GelDoc XR+ ImageLab (Bio-Rad Laboratories, Inc., Hercules, CA, USA).

### 2.5. Co-Immunoprecipitation (Co-IP)

The clarified cell lysates of 1 × 10^7^ Caco-2 cells transfected with pcDNA3.1-HBoV1VP3-StrepII or pcDNA3.1-HBoV2VP3-StrepII were incubated with MagStrep Strep-Tactin^TM^ XT Beads (Fisher Scientific, Hampton, NH, USA) in IP buffer (50 mM Tris-HCl pH 7.4, 150 mM NaCl, 1 mM EDTA, 1% Triton X-100, and protease inhibitor cocktail) at 4 °C under gentle rotation overnight. Cells transfected with empty pcDNA3.1 vector served as the negative control. The beads were then washed extensively with lysis buffer to remove non-specifically bound proteins. Then protein–protein complexes were later subjected to SDS-PAGE gel. The excised gel with expected protein bands was retained, dehydrated with acetonitrile, and subsequently reduced with dithiothreitol (DTT) and alkylated with iodoacetamide (IAA). The gel pieces were then digested with trypsin at 37 °C overnight. To purify and desalt the digested peptides, the samples were processed using a C18 solid-phase extraction (SPE) cartridge (Supelco, Bellefonte, PA, USA). The cartridge was activated with acetonitrile, equilibrated with 2% acetonitrile containing 0.1% formic acid, and then loaded with the peptide mixture. After extensive washing with the equilibration solution to remove salts, the peptides were eluted using 50% acetonitrile containing 0.1% formic acid. The combined eluted peptides were vacuum-centrifuged to dryness and stored at −80 °C.

### 2.6. Liquid Chromatography–Tandem Mass Spectrometry (LC-MS)

The lyophilized peptides were reconstituted in 0.1% formic acid and separated using an EASY-nLC 1200 system (Thermo Fisher Scientific, Waltham, MA, USA). Separation was performed on a reverse-phase C18 capillary column (150 μm × 150 mm, 1.9 μm) with a mobile phase consisting of solvent A (0.1% formic acid in water) and solvent B (0.1% formic acid in 80% acetonitrile). A 90 min gradient from 6% to 42% solvent B was applied at a flow rate of 0.6 μL/min. The eluted peptides were analyzed using an Orbitrap Exploris^TM^ 240 mass spectrometer (Thermo Fisher Scientific, USA) operated in data-dependent acquisition mode. Full MS scans were acquired at a resolution of 60,000, and MS scans were performed at a resolution of 15,000.

For the differentially interacting proteins identified by LC-MS, their biological significances were evaluated by the Gene Ontology (GO) and Kyoto Encyclopedia of Genes and Genomes (KEGG) pathway enrichment analyses.

### 2.7. In Vitro Pull-Down Assays

In the in vitro pull-down assays to determine the interacted proteins, 1 μg of the (Glutathione S-transferase) GST-tagged commercial host proteins expressed in prokaryotic cells were immobilized on glutathione Sepharose beads. Then, 1 μg of the HBoV1 and HBoV2 VP3 expressed in BL21 (DE3) *E. coli* were added to the mixture [18]. In the assays, negative controls were included. HBoV1 and HBoV2 VP3 were incubated with GST-bound beads without GST-tagged host proteins. After washing four times with wash buffer, the bound proteins were eluted and analyzed by WB.

### 2.8. Structural Prediction and Molecular Docking Analysis

The tertiary structures of HBoV1 and HBoV2 VP3 monomers were predicted using AlphaFold2. For each genotype, the model with the highest PLDDT (predicted local distance difference test) score was selected as the representative structure for subsequent analyses [22]. Molecular docking simulations were then performed to predict the most plausible complex structures between each VP3 variant and its respective host protein partner. These simulations were conducted using the HDOCKlite v1.1 local server. The docking algorithm sampled numerous binding conformations, which were ranked using an integrated scoring function. The optimal docking pose for each VP3–host protein pair was selected based on a combination of docking score and confidence score. Finally, the intermolecular interactions stabilizing the predicted complexes—including hydrophobic contacts, hydrogen bonds, and electrostatic forces—were analyzed in detail.

### 2.9. Statistical Analysis

Statistical analyses were performed with GraphPad Prism version 10.0 (GraphPad Software, La Jolla, CA, USA). Non-normally distributed data were expressed as median with interquartile range (IQR) and were analyzed using the Mann–Whitney U test. A two-sided *p*-value < 0.05 was considered statistically significant.

## 3. Results

### 3.1. Host Proteins Specifically Interacting with HBoV1 or HBoV2 VP3

**Figure 1:**
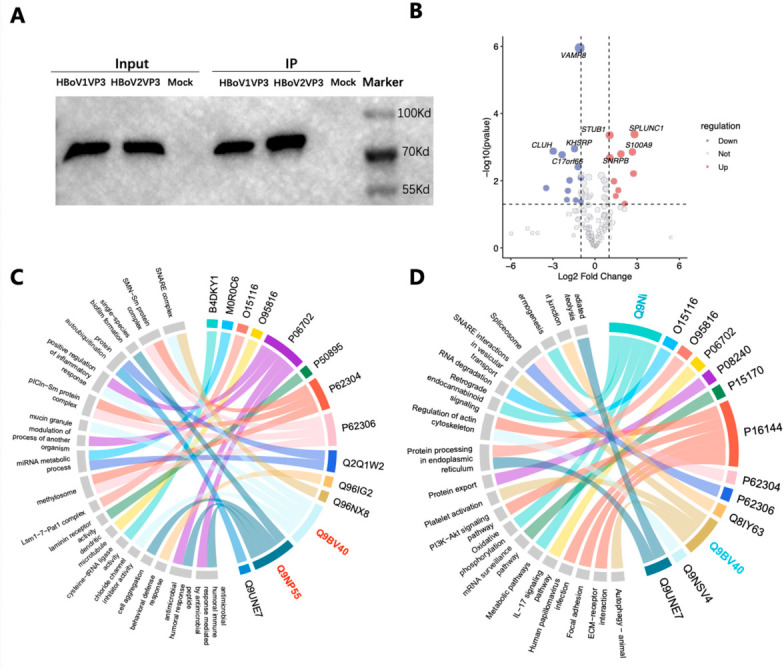
IP-MS combined with GO and KEGG analysis to identify host proteins specifically interacting with HBoV1 or HBoV2 VP3. (**A**) Immunoblot analysis of StrepII-tagged HBoV1-VP3 (1-VP3) and HBoV2-VP3 (2-VP3) expressed in Caco-2 cells. Input, whole-cell lysates; IP, StrepII-tag pull-down eluates. The vector-transfected (pcDNA3.1) lysate serves as a negative control (Mock). The recombinant VP3 proteins migrated at approximately 70 kDa. (**B**) Volcano plot analysis of proteins differentially interacting with HBoV1-VP3 (group 1) versus HBoV2-VP3 (group 2). Horizontal axis, log 2 (fold change); vertical axis, −log10(*p*-value). Dotted line indicates the threshold for statistical significance (adjusted *p*-value < 0.05). (**C**) GO functional enrichment analysis of proteins selectively bound to HBoV1- or HBoV2-VP3. Terms are ranked by enrichment score. (**D**) KEGG pathway enrichment analysis. Bubble size corresponds to the number of enriched proteins; color scale indicates the −log10 (*p*-value) of enrichment.

In WB analysis, specific bands of VP3 at the expected molecular weight of approximately 70 kDa were detected from Caco-2 cells transfected with the pcDNA3.1-HBoV1 VP3 or pcDNA3.1-HBoV2 VP3 plasmid, which were also shown in the eluted Co-IP products used to in vitro pull down assay for searching the potential host interaction proteins (Figure 1A, Supplementary Table S1).

Using the subtraction of proteins presenting in cells transfected with pcDNA3.1 vector as the mock control to eliminate non-specific binders, a refined set of 276 putative interacting proteins with high-confidence was retained for subsequent analysis. High-confidence interactors were defined as those with ≥2 unique peptides and a fold change > 2 relative to mock controls. Of these, 148 eukaryotic host proteins were identified. According to the binding affinities of these 148 host proteins with HBoV1 and HBoV2 VP3 quantified, 50 host proteins displayed ≥10-fold differentially interactions with HBoV1 and HBoV2 VP3 (adjusted *p* ≤ 0.05, Benjamini–Hochberg FDR correction) (Figure 1B) (Supplemental Table S1).

By integrating the results of GO and KEGG enrichment analyses, seven proteins containing >10 unique peptides and showing ≥10-fold differential binding were selected for further biological analysis to confirm their potential relevance to tissue tropism. Among them, short palate, lung and nasal epithelium carcinoma associated 1 (SPLUNC1) showed binding affinities to HBoV1 VP3 that were more than 10 times higher than those to HBoV2 VP3 (*p* = 0.000) in quantitative analysis. And a core component of the soluble N-ethylmaleimide-sensitive factor A attachment protein receptor (SNARE) complex VAMP8, which participated in vesicle fusion, showed binding affinities to HBoV2 VP3 that were more than 10 times higher than those to HBoV1 VP3 (*p* = 0.000) (Figure 1C,D) (Supplemental Table S2).

### 3.2. Validation of the Direct Interactions of HBoV1 and HBoV2 VP3 with Host Proteins SPLUNC1 or VAMP8 In Vitro

**Figure 2:**
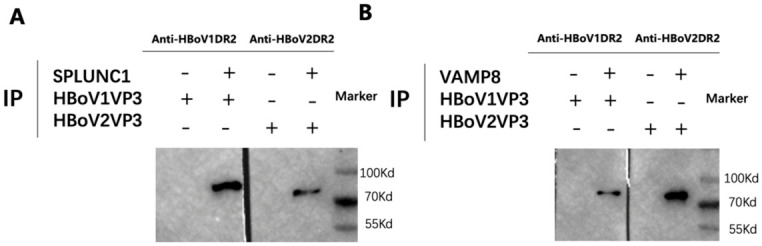
In vitro binding assays of HBoV1 and HBoV2 VP3 with SPLUNC1 and VAMP8. (**A**) Pull-down assay of HBoV1 and HBoV2 VP3 with SPLUNC1. Purified VP3 proteins were incubated with GST-SPLUNC1 immobilized on glutathione Sepharose beads. Bound proteins were immunoblotted with anti-HBoV1DR2 or anti-HBoV2DR2 polyclonal antibodies, respectively [18]. (**B**) Pull-down assay of HBoV1 and HBoV2 VP3 with VAMP8, performed similarly using GST-VAMP8 beads. The predicted molecular weight of the recombinant HBoV1/2 VP3 proteins is 70 kDa.

To confirm the interaction of the host protein SPLUNC1 with HBoV1 and HBoV2 VP3, the pull-down assay was performed in vitro. HBoV1 VP3 showed no binding to the GST beads in the control group but was specifically pulled down by the GST beads with SPLUNC1, which confirmed the direct interaction of HBoV1 VP3 with SPLUNC1. However, low binding signal was detected in the interaction of HBoV2 VP3 with GST-SPLUNC1. In another pull-down assay conducted in vitro to confirm the interaction of the host protein VAMP8 with HBoV1 and HBoV2 VP3, HBoV2 VP3 showed no binding to GST beads in the control group but was specifically pulled down by GST beads with VAMP8, while HBoV1 VP3 showed very low signal in binding with GST-VAMP8 (Figure 2A).

### 3.3. Docking Analysis Between VP3 of HBoV1 or HBoV2 and Host Proteins

**Figure 3:**
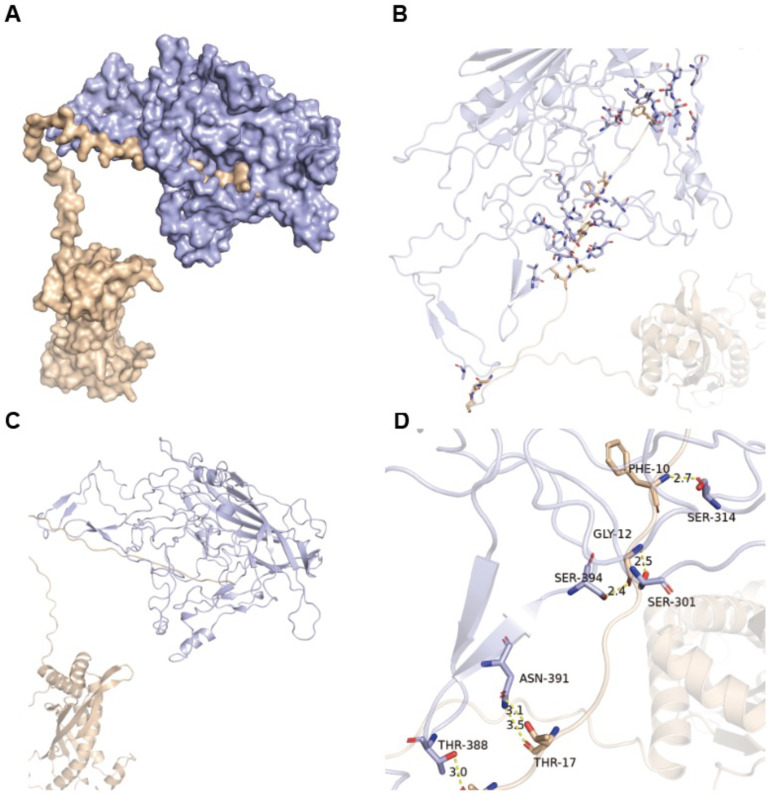
Hydrophobic interface and hydrogen bonding between HBoV1 VP3 (purple) and SPLUNC1 (yellow). (**A**) Overall binding interface between HBoV1 VP3 and SPLUNC1. (**B**) Close-up view of hydrogen bonding interactions involving SER-314, SER-304, and SER-301. (**C**) Detailed interactions of ASN-391 and THR-388 at the interface. (**D**) Local environment of THR-17 and its potential role in stabilizing the complex.

The molecular docking analysis was performed to reveal the interaction modes between HBoV1 VP3 and host protein SPLUNC1. The dock score for the complex HBoV1 VP3 with SPLUNC1 was 2123.322. The interaction was primarily stabilized by an extensive hydrophobic interface involving residues from both proteins. Additionally, specific hydrogen bonds were identified between HBoV1 VP3 (THR-388, ASN-391, SER-394, SER-301, and SER-314) and SPLUNC1 (ALA-19, THR-17, GLY-12, and PHE-10), with bond lengths ranging from 2.4 to 3.5 Å (Figure 3). A detailed list of all residues involved in hydrophobic interactions is provided in Supplementary Table S3. Notably, mapping the interaction interface residues onto the previously defined divergent regions (DR1–4) of HBoV VP3 revealed that residues involved in HBoV1 VP3-SPLUNC1 binding (including SER-301, ALA-302, and ASN-276) are predominantly located within or adjacent to the DR2 region, which has been shown to be surface-exposed and genotype-specific [18].

**Figure 4:**
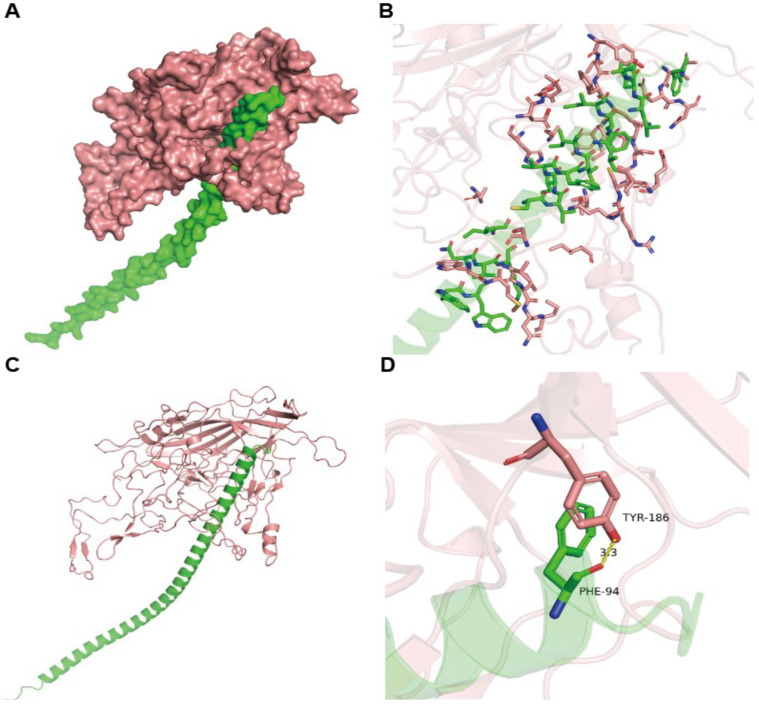
Hydrophobic interface and hydrogen bonding between HBoV2 VP3 (pink) and VAMP8 (green). (**A**) Overall binding interface between HBoV2 VP3 and VAMP8. (**B**) Detailed view of hydrophobic packing involving TYR-186 and PHE-94. (**C**) Hydrogen bond network formed by surrounding polar residues at the interface. (**D**) Surface representation showing the complementarity of the binding groove.

The molecular docking analysis was similarly performed to reveal the interaction mode between HBoV2 VP3 and the host protein VAMP8. The dock score for the HBoV2 VP3–VAMP8 complex was 2261.084. The interaction was primarily stabilized by an extensive hydrophobic interface formed by residues from both proteins. In addition, a specific hydrogen bond was identified between TYR-186 of HBoV2 VP3 and PHE-94 of VAMP8, with a bond length of 3.3 Å (Figure 4). A detailed list of all residues involved in hydrophobic interactions is provided in Supplementary Table S3. The HBoV2 VP3 residues, critical for VAMP8 interaction (TYR-186, ASP-173, ALA-168), map to the DR1 and DR3 regions. This correlation between genotype-specific sequence divergence and differential host protein binding provides structural support for the observed interaction specificity.

Quantitative analysis of the docking models revealed that the HBoV1 VP3-SPLUNC1 complex has a calculated binding free energy (ΔG) of −12.3 kcal/mol and a buried surface area of 1856 Å^2^, while the HBoV2 VP3-VAMP8 complex has a ΔG of −14.7 kcal/mol and a buried surface area of 2104 Å^2^, suggesting that both interactions are energetically favorable. A detailed comparison of binding parameters is provided in Supplementary Table S4.

## 4. Discussion

Different genotypes of HBoVs sharing a high degree of genomic homology displayed distinct clinical manifestations, which implied their different tissue tropisms. Epidemiologically, HBoV1 is primarily linked to respiratory tract infections, whereas HBoV2 is predominantly associated with gastroenteritis and is frequently detected in fecal samples. The viral capsid protein VP3, as the principal structural component, mediates the critical initial interaction between the virus and the host cell. To elucidate the molecular basis of this tissue specificity, a comparative Co-IP/MS approach to profile the differential host protein interactomes of HBoV1 VP3 and HBoV2 VP3 was employed. The analysis identified SPLUNC1 and VAMP8 as host proteins that exhibited specific binding to HBoV1 VP3 and HBoV2 VP3, respectively.

Interaction profiling confirmed SPLUNC1 as a highly specific interactor of HBoV1 VP3. SPLUNC1 is a secreted innate immune protein and predominantly expressed in the upper and lower respiratory tracts in abundance. It plays a crucial role in mucosal defense by promoting mucociliary clearance, disrupting bacterial biofilms, and modulating epithelial ion channel activity [23,24]. The specific binding of HBoV1 VP3 to this airway-specific and enriched factor, as demonstrated by pull-down assays, strongly suggests that SPLUNC1 may function as a key attachment factor or a component of a host receptor complex, facilitating the efficient docking of HBoV1 to the respiratory epithelium. The strategy—exploiting tissue-enriched innate immune factors for cellular entry—is well-documented among other respiratory viruses. Conversely, the negligible binding of HBoV2 VP3 to SPLUNC1 provides molecular support for the limited respiratory tropism observed for HBoV2.

In contrast, the SNARE protein VAMP8 (Vesicle-Associated Membrane Protein 8) was identified as a preferential and specific interactor of HBoV2 VP3 [25,26]. VAMP8 is a key regulator of membrane fusion events, including those involved in the autophagy and lysosomal pathways. Notably, VAMP8 plays an initial role in the exocytosis of MUC2 mucin granules in intestinal goblet cells, a process critical for maintaining the protective intestinal mucus barrier [27,28]. The robust and specific interaction between HBoV2 VP3 and VAMP8 suggests a dual-pathway mechanism that may facilitate intestinal infection. First, HBoV2 could exploit VAMP8 to promote endosomal escape or intracellular trafficking within enterocytes, a mechanism consistent with VAMP8-mediated entry observed in other viruses. Second, this interaction may modulate mucin secretion, thereby disrupting the mucosal barrier to enhance viral access to the underlying epithelium or to aid in evading initial immune defenses [29]. In comparison, the weak interaction observed between HBoV1 Vp3 and VAMP8 indicates that this pathway is not a primary entry mechanism for the respiratory genotype.

Predicted tertiary structures of the VP3 proteins and molecular docking simulations provided structural support for these differential binding events. The docking models indicated that stable complexes are formed primarily through extensive hydrophobic interfaced, supplemented by specific hydrogen bonds. In the SPLUNC1-HBoV1 VP3 complex, several hydrogen bonds are formed between residues of HBoV1 VP3 (e.g., Thr-388, Asn-391, Ser-394, Ser-301, and Ser-314) and SPLUNC1 (e.g., Ala-19, Thr-17, Gly-12, and Phe-10). The VAMP8-HBoV2 VP3 complex features a broad hydrophobic interface and is further stabilized by a specific hydrogen bond between Tyr-186 on HBoV2 VP3 and Phe-94 on VAMP8. The docking models further revealed that the interaction interfaces map to previously identified divergent regions (DR1–4) of VP3, which are known to be surface-exposed and genotype-specific. This correlation between sequence divergence and differential binding specificity provides a structural basis for the observed dichotomy in host protein interactions and supports the functional relevance of these regions in determining tissue tropism.

The specific binding of HBoV1 VP3 to this airway-specific and enriched factor strongly suggests that SPLUNC1 may function as a key attachment factor or a component of a host receptor complex, facilitating the efficient docking of HBoV1 to the respiratory epithelium. Given that SPLUNC1 is a secreted protein present in airway surface liquid [30], it may act at the earliest stage of infection by concentrating viral particles on the epithelial surface prior to engagement with a secondary internalization receptor. This strategy—exploiting tissue-enriched innate immune factors for cellular entry—is well-documented among other respiratory viruses, including influenza A virus and respiratory syncytial virus. Conversely, the negligible binding of HBoV2 VP3 to SPLUNC1 provides molecular support for the limited respiratory tropism observed for HBoV2.

Regarding VAMP8, its role as a SNARE protein mediating membrane fusion suggests multiple potential functions during HBoV2 infection. First, HBoV2 could exploit VAMP8 to promote endosomal escape or intracellular trafficking within enterocytes, as VAMP8 is known to regulate fusion events along the autophagic and endocytic pathways [31]. This mechanism would be consistent with VAMP8-mediated entry observed in other viruses, including West Nile virus [27]. Second, given VAMP8’s critical role in MUC2 mucin granule exocytosis in intestinal goblet cells [28,29], HBoV2 VP3 binding to VAMP8 may modulate mucin secretion, thereby disrupting the protective mucosal barrier to enhance viral access to the underlying epithelium or to aid in evading initial immune defenses. This dual-pathway mechanism—directly facilitating viral entry while indirectly compromising barrier function—would be particularly advantageous for an enteric pathogen.

This study has several limitations. First, both the initial Co-IP/MS screening and subsequent validation experiments were primarily performed in Caco-2 cells, an intestinal epithelial cell line. While this background is appropriate for HBoV2-related findings, it may not fully recapitulate the respiratory environment for HBoV1. To partially address this, we will verify the HBoV1 VP3-SPLUNC1 interaction in A549 lung epithelial cells in the future. Second, while we provide robust evidence for direct physical interactions between HBoV1 VP3 and SPLUNC1, and between HBoV2 VP3 and VAMP8, the functional relevance of these interactions during the viral life cycle has yet to be fully determined. Future studies employing genetic manipulation (e.g., CRISPR/Cas9-mediated knockout or siRNA-mediated knockdown of SPLUNC1 in airway epithelial cells and VAMP8 in intestinal epithelial cells) followed by infection with recombinant HBoV1 or HBoV2 will be necessary to establish whether these host factors are required for efficient viral entry or post-entry processes, and whether they indeed determine tissue tropism. Third, this study utilized VP3 expressed alone as the bait for host protein interaction screening. While VP3 is the major capsid component and can self-assemble into VLPs, native HBoV virions contain VP1 and VP2 in addition to VP3 at a ratio of approximately 1:1:10. The presence of VP1 and VP2, particularly their unique N-terminal extensions, may influence the surface exposure of VP3 epitopes or create additional interaction interfaces. Therefore, the host protein interactions identified in this study represent a subset of potential interactions mediated by VP3 in the context of VP3-only assemblies. Future studies using native virions or full capsids containing all three VPs are warranted to comprehensively map the virus–host interaction.

## 5. Conclusions

This study provides molecular evidence that the distinct tissue tropisms of HBoV1 and HBoV2 are mediated by specific interactions between their respective VP3 capsid proteins and the host factors SPLUNC1 and VAMP8. These virus–host interactions not only offer a mechanistic basis for the observed tropism dichotomy but also define promising molecular targets for future antiviral strategies.

## Data Availability

The data supporting the findings of this study are available from the corresponding author upon reasonable request.

## Supplementary Materials

The following supporting information can be downloaded at https://www.mdpi.com/article/10.3390/pathogens15050486/s1: Table S1: Significant differently interaction protein information; Table S2: Host proteins specifically interacting with HBoV1 or HBoV2 VP3 screened by IP-MS.; Table S3: Key residues involved in the interactions between VP3 and host proteins as predicted by molecular docking.; Table S4: Quantitative comparison of docking results for VP3-host protein complexes.
