# Supplementary material for: Differential Interactions of Tissue-Restricted Host Proteins SPLUNC1 and VAMP8 with VP3 of Human Bocaviruses 1 and 2
Source: Pathogens. 2026 May 1;15(5):486. doi: 10.3390/pathogens15050486 (PMC13209220; doi:10.3390/pathogens15050486)
Supplement: Supplementary file 1 [file pathogens-15-00486-s001.zip › pathogens-4247347-supplementary.pdf]

Supplementary Table S1. Significant differently interaction protein information.

Protein IDs	Fasta headers	Unique peptides	Mol. weight [kDa]
Q9NSV4	sp|Q9NSV4|DIAP3_HUMAN	7	136.92
Q96NX8	tr|Q96NX8|Q96NX8_HUMAN	2	23.021
Q2Q1W2	sp|Q2Q1W2|LIN41_HUMAN	3	93.384
G3V4P8	tr|G3V4P8|G3V4P8_HUMAN	2	17.512
R4GMW4	tr|R4GMW4|R4GMW4_HUMAN	2	8.6607
**A0A7P0TAQ9**	**tr|A0A7P0TAQ9|A0A7P0TAQ9_HUMAN**	**14**	**87.996**
E9PKK8	tr|E9PKK8|E9PKK8_HUMAN	2	19.288
A0A6Q8PH33	tr|A0A6Q8PH33|A0A6Q8PH33_HUMAN	2	14.361
I3L0H8	tr|I3L0H8|I3L0H8_HUMAN	4	50.519
E9PK54	tr|E9PK54|E9PK54_HUMAN	1	19.955
C9JFK9	tr|C9JFK9|C9JFK9_HUMAN	2	34.759
F5GXM3	tr|F5GXM3|F5GXM3_HUMAN	2	16.11
P15170	sp|P15170|ERF3A_HUMAN	2	55.755
**M0R0C6**	**tr|M0R0C6|M0R0C6_HUMAN**	**27**	**76.65**
F5H3C5	tr|F5H3C5|F5H3C5_HUMAN	2	12.13
P08240	sp|P08240|SRPRA_HUMAN	2	69.81
O15116	sp|O15116|LSM1_HUMAN	1	15.179
**O95816**	**sp|O95816|BAG2_HUMAN**	**19**	**23.772**
B4DKY1	tr|B4DKY1|B4DKY1_HUMAN	2	84.277
E9PMK8	tr|E9PMK8|E9PMK8_HUMAN	1	28.668
P15374	sp|P15374|UCHL3_HUMAN	2	26.182
G5E9L9	tr|G5E9L9|G5E9L9_HUMAN	1	76.148
P62306	sp|P62306|RUXF_HUMAN	2	9.7251
Q8N0Z8	sp|Q8N0Z8|PUSL1_HUMAN	2	33.232
**Q9BV40**	**sp|Q9BV40|VAMP8_HUMAN**	**12**	**11.438**
M0R2P6	tr|M0R2P6|M0R2P6_HUMAN	2	73.707
O00483	sp|O00483|NDUA4_HUMAN	2	9.3697
A0A088AWN3	tr|A0A088AWN3|A0A088AWN3_HUMAN	3	237.91
A0A0U1RRL8	tr|A0A0U1RRL8|A0A0U1RRL8_HUMAN	3	88.051
D6RGE2	tr|D6RGE2|D6RGE2_HUMAN	3	20.239
C9JLF4	tr|C9JLF4|C9JLF4_HUMAN	1	12.421
**Q9UNE7**	**sp|Q9UNE7|CHIP_HUMAN**	**14**	**34.856**
**A0A494C0R8**	**tr|A0A494C0R8|A0A494C0R8_HUMAN**	**23**	**150.46**
Q96IG2	sp|Q96IG2|FXL20_HUMAN	3	48.423
P50895	sp|P50895|BCAM_HUMAN	5	67.404
A0A087WW76	tr|A0A087WW76|A0A087WW76_HUMAN	2	155.98
P62304	sp|P62304|RUXE_HUMAN	1	10.803
O60664	sp|O60664|PLIN3_HUMAN	6	47.074
P06702	sp|P06702|S10A9_HUMAN	5	13.242
Q8N283	sp|Q8N283|ANR35_HUMAN	2	109.96
Q9NRP0	sp|Q9NRP0|OSTC_HUMAN	3	16.829
I3L1H9	tr|I3L1H9|I3L1H9_HUMAN	1	7.3695
I3L4G8	tr|I3L4G8|I3L4G8_HUMAN	4	20.167
J3KN01	tr|J3KN01|J3KN01_HUMAN	1	205.64
Q9H3K2	sp|Q9H3K2|GHITM_HUMAN	3	37.205
**Q9NP55**	**sp|Q9NP55|SLPUNC_HUMAN**	**16**	**26.712**

A0A140T937	tr|A0A140T937|A0A140T937_HUMAN	0	38.331
E9PL71	tr|E9PL71|E9PL71_HUMAN	1	20.819
P16144	sp|P16144|ITB4_HUMAN	2	202.16
Q8IY63	sp|Q8IY63|AMOL1_HUMAN	3	106.57

Supplementary Table S2. Host proteins specifically interacting with HBoV1 or HBoV2 VP3 screened by IP-MS.

Name of proteins	Abbreviations	Functions
DnaJ homolog subfamily C member 10	DNAJC10	An ER-localized protein involved in ER-associated degradation (ERAD), facilitating disulfide bond reduction in misfolded proteins for cytosolic degradation. Interacts with Hsp70 via its J-domain and contributes to ER protein quality control.
KH-type splicing regulatory protein	KHSRP	An RNA-binding protein regulating mRNA splicing, stability, and miRNA processing. Modulates immune and inflammatory responses through pathways including JAK-STAT.
BAG family molecular chaperone regulator 2	BAG2	A co-chaperone binding Hsp70/Hsc70 to stimulate nucleotide exchange and inhibit CHIP-mediated ubiquitination. Regulates protein folding-degradation balance and cellular stress response.
**Vesicle-associated membrane protein 8**	**VAMP8**	**A SNARE protein mediating membrane fusion in autophagy and mucin secretion. Supports antiviral immunity and mucosal barrier function via MUC2 exocytosis.**
E3 ubiquitin-protein ligase CHIP	STUB1	A chaperone-dependent E3 ubiquitin ligase targeting misfolded proteins for degradation. Modulates inflammation and T-cell regulation through ubiquitination.
Clustered mitochondria protein homolog	CLUH	An RNA-binding protein regulating transport and translation of nuclear-encoded mitochondrial mRNAs. Essential for mitochondrial biogenesis and function.
**Short Palate, Lung and Nasal Epithelium Carcinoma Associated 1**	**SPLUNC1**	**An airway innate immune protein that disrupts bacterial biofilms, inhibits ENaC activation, and supports mucociliary clearance and antiviral defense.**

Supplementary Table S3. Key residues involved in the interactions between VP3 and host proteins as predicted by molecular docking

Complex	Protein	Residues Involved in Hydrophobic Interactions	Residues Involved in Hydrogen Bonds	Bond Length (Å)
**HBoV1 VP3 – SPLUNC1**	HBoV1 VP3	TRP-315, VAL-317, PHE-337, PRO-340, PHE-431, TYR-351, LEU-299, PRO-297, LEU-397, ALA-313, GLY-298, ASP-338, ALA-302, ASP-395, LEU-300, ASN-167, GLN-303, GLU-75, ALA-278, TYR-192, SER-91, PRO-93, TYR-190, ALA-168, THR-92, GLY-191, ASN-276, PHE-188, ASN-68, ASP-173, VAL-90	THR-388<br>ASN-391<br>SER-394<br>SER-301<br>SER-314	3.0<br>3.1<br>3.5<br>2.4<br>2.5
	SPLUNC1	ILE-8, LEU-7, TYR-11, LEU-14, LEU-13, LEU-24, PHE-2, GLY-22, GLY-23	ALA-19<br>THR-17<br>GLY-12<br>PHE-10	2.7<br>3.0<br>3.1<br>3.5
**HBoV2 VP3 – VAMP8**	HBoV2 VP3	PRO-321, ASN-320, TRP-315, VAL-319, ILE-196, MET-316, VAL-318, SER-335, VAL-90, TYR-190, GLY-191, GLN-189, SER-91, ARG-88, CYS-89, TYR-192, THR-92, GLN-87, ILE-193, PHE-188, LEU-187, GLU-174, ILE-77, TYR-186, ASP-173, ASP-175, ALA-168, PRO-420, GLU-75, HIS-170, GLN-67, ASN-167, ASN-68, ILE-66	TYR-186	3.3
	VAMP8	TRP-71, TRP-70, LYS-75, ASN-73, VAL-74, ILE-77, PHE-90, ILE-86, CYS-81, VAL-82, PHE-85, ILE-91, LEU-89, ALA-95, PHE-94, ILE-87, VAL-84, MET-76, ILE-83, LEU-93, PHE-99	PHE-94	3.3

Supplementary Table S4. Quantitative comparison of docking results for VP3-host protein complexes

Parameter	HBoV1 VP3-SPLUNC1	HBoV2 VP3-VAMP8
Dock score	2123.322	2261.084
Binding free energy (ΔG, kcal/mol)	-12.3	-14.7
Buried surface area (Å^2)	1,856	2,104
Number of hydrogen bonds	5	1
Number of hydrophobic contacts	38	42
